# *Mycobacterium tuberculosis acg* Gene Is Required for Growth and Virulence In Vivo

**DOI:** 10.1371/journal.pone.0020958

**Published:** 2011-06-08

**Authors:** Yanmin Hu, Anthony R. M. Coates

**Affiliations:** Division of Clinical Sciences, Infection and Immunity Research Centre, St. George's University of London, London, United Kingdom; Hopital Raymond Poincare - Universite Versailles St. Quentin, France

## Abstract

*Mycobacterium tuberculosis* *dosRS* two-component regulatory system controls transcription of approximately 50 genes including *hspX*, *acg* and *Rv2030c,* in response to hypoxia and nitric oxide conditions and within macrophages and mice. The *hspX* lies between *acg* and *Rv2030c*. However, the functions of the *dosR* regulated genes in vitro and in vivo are largely unknown. Previously, we demonstrated that deletion of *hspX* gene produced a mutant which grew faster in macrophages and in mice. In this study, we attempted to determine the functions of *acg* and *Rv2030c* by gene inactivation. We demonstrate that *Rv2030c* is dispensable for virulence and growth. However, deletion of *acg* produced a mutant which is attenuated in both resting and activated macrophages and in acute and persistent murine infection models. Surprisingly, deletion of *acg* did not compromise the viability of the mutant to nitrosative and oxidative stresses in vitro and in vivo. In addition, when the WT and the *acg* mutants were treated with antibiotics such as the prodrugs nitrofurantoin and nitrofuran, the *acg* mutant became more sensitive than the WT strain to these drugs. This suggests that Acg may not function as a nitroreductase. These data indicate that *acg* encodes an essential virulence factor for *M. tuberculosis* and enables it to grow and survive in macrophages and in mouse organs.

## Introduction

Tuberculosis (TB) remains one of the major infectious diseases, causing 8% of all deaths worldwide [1]. Currently, over two billion people are infected with the causative agent, *Mycobacterium tuberculosis*. Once infected, an individual becomes a life-long carrier, with a 5–10% lifetime risk of contracting acute disease [2], [3]. A major biological question is the mechanisms by which *M. tuberculosis* controls its virulence. Clearly, a latent state plays an important role in which it needs to be non-virulent and non-transmissible.

In experimental models, the latent state is thought to be regulated by hypoxia [4] whose response in the bacterium is controlled by the *dosRS* two-component regulatory system (2CR) [5], [6]. The *dosRS* system controls the transcription of about 50 genes under hypoxic conditions and in response to nitric oxide [5], [7]. Recent work demonstrated that the *dosR* regulon is regulated by carbon monoxide which is produced by *M. tuberculosis* infected macrophages and other in vitro stress conditions [8], [9], [10]. Also, many genes in the *dosR* regulon show increased expression in murine macrophages and in murine lung tissues [9], [11], [12] and these genes may be involved in survival and persistence of the bacterium in vivo.

**Figure 1:**
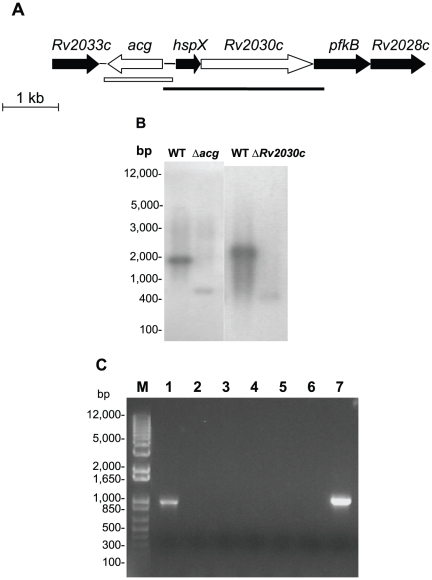
Construction of the *M. tuberculosis acg* and *Rv2030c* mutants. A. Genomic context of the genes upstream and downstream of *hspX* gene. The open arrows indicate the *acg* or *Rv2030c* gene which was deleted in the mutant. The open box and the line show the location of the fragments used for complementation of YHΔ*acg* or YH*Rv2030c*, respectively. These were also used as probes for southern blotting analysis. B. Southern blotting analysis to confirm the deletion of the genes. DNA from WT and YHΔ*acg* or YH*Rv2030c* was digested with *Pvu* II and *Btr* I, respectively and hybridised with the probes which used to make the complemented constructs. bp, molecular weight marker (Invitrogen). The experiments were repeated twice, with identical results. C. Detection of *acg* mRNA in WT and YHΔ*acg* by RT-PCR. RNA was extracted from 7 day-old cultures. M. molecular weight marker (Invitrogen). 1. RNA extracted from the WT strain, 2. DNase I-treated RNA isolated from the WT culture but no RT enzyme. 3. PCR negative control with each primer and water instead of template. 4. RNA extracted from YHΔ*acg.* 5. DNase I-treated RNA isolated from the *acg* mutant culture but no RT enzyme. 6. PCR negative control with each primer and water instead of template. 7. PCR positive control with each primer and *M. tuberculosis* WT DNA.

Most of the genes controlled by *dosR* have unknown or predicted functions. For example, *narX* [13], is predicted to encode a nitrate reductase which may help the bacterium to adapt to hypoxia. Protection against nitrogen stress is predicted [13] to be mediated by the nitroreductase genes *acg* (Rv2032), *Rv3127* and *Rv3131*. It is thought that *hspX* [14], [15] may be involved in latency. A recent study using bioinformatic analysis showed that the *dosR* regulated genes may be involved in carbohydrate and fatty acid metabolism [16]. It is important to investigate if the genes within the *dosRS* regulon exhibit similar or diverse functions in vivo as this will help us to understand the biological relevance of the class of the genes and their roles in survival and persistence of the organism in human infection. Previously, studies using high density mutagenesis showed that most of the *dosR* regulated genes were not essential for growth [17]. In the past, with the aim of dissecting the potential mechanism which underlies the *dosR* regulatory system, *dosR* has been inactivated in *M. tuberculosis.* Counter intuitively, this produced a mutant that was hypervirulent in activated macrophages and in murine tuberculosis [18]. We made an unmarked *hspX* deletion mutant of *M. tuberculosis* [19] which showed faster growth in macrophages and in mice. This was also confirmed by another study of *hspX* gene knock out [20]. However, later studies produced *dosR* deletion mutants which either showed an attenuated phenotype in guinea pigs, mice and rabbits [21] or had no growth deficit in mice [22]. This raised the question as to the in vivo functions of the genes adjacent to the *hspX* gene. It has been shown previously that a *hspX* mutant in which *hspX* was replaced by a hygromycin-resistance gene [14] was attenuated in a macrophage model, suggesting that it is required for virulence. We hypothesised the reason for the contradictory findings between the hygromycin-resistance *hspX* gene deletion [14] and our unmarked deletion [19] was that the hygromycin-resistance gene deletion mutant had alterations in the genes which are immediately adjacent to *hspX*, namely *acg* which lies upstream, and *Rv2030c* which is downstream (see Fig. 1). The *hspX* and *acg* promoters which express divergently share the intergenic region [13], [23]. Interruption of this intergenic region may affect expression of both genes [14]. It is particularly interesting to investigate the function of the *acg* gene which has been suggested to encode a putative classical nitroreductase [13], [24]. *acg* is one of the most upregulated genes in the *dosR* regulon. The expression of *acg* was found to be coregulated with that of the *hspX* gene under low O_2_ conditions, within macrophages, especially activated macrophages and in mice [11], [13]. It has been suggested that *acg* might play an important role in *M. tuberculosis* detoxification of nitrogen intermediates [13]. The downstream gene *Rv2030c* of unknown function is co-transcribed with *hspX* [15].

In this paper, we deleted *acg* and *Rv2030c.* Deletion of *acg* led to an attenuated mutant in macrophage infection and in vivo acute and persistent murine models. The *M. tuberculosis* mutant lacking the *Rv2030c* gene was identical to the parent strain. This suggests that the *hspX* and its adjacent genes although regulated by DosR are functionally distinct.

## Results

### Construction of *acg* and *Rv2030c* mutants of *M. tuberculosis*

Previously, we showed that deletion of *hspX* results in rapid growth of the organism *in vivo.* As seen in Fig. 1A, *hspX* is the first gene of a possible operon with several downstream genes *RV2030c*, *pfkB* and *Rv2028c*. The intergenic region between each gene only contains a few base pairs of nucleotides. Upstream of *hspX* is *acg* which translates in the opposite direction. In order to verify the essentiality of the genes adjacent to *hspX* in vivo, we made two *M. tuberculosis* mutants which deleted *acg* or *Rv2030c* using a two-step mutagenesis strategy [25]. The generation of the mutant strain was confirmed by PCR (data not shown) and Southern blotting analysis (Fig. 1B). The expression of *acg* gene (Fig. 1C) and *Rv2030c* (data not shown) was also confirmed by RT-PCR. The *M. tuberculosis* strains lacking these genes were defined as YHΔ*acg* and YHΔ*Rv2030c*, respectively. Both sequences of the PCR products amplified from WT and the mutants were analysed by DNA sequencing to confirm the deletion of these two genes.

The deletion of *acg* or *Rv2030c* was complemented by the introduction of the integrating plasmid pUC-Gm-Int containing the native gene and promoter into the mutant. This suicide plasmid contains a gene which encodes the integrase of phage L5, the *attP* site and a gentamicin resistant marker cassette. The gene integration into the chromosome of the mutant was selected for gentamicin resistance. The complemented strains were called YH*acg*Comp and YH*Rv2030c*Comp.

### Growth and survival of YHΔ*acg* and YHΔ*Rv2030c* in macrophages

**Figure 2:**
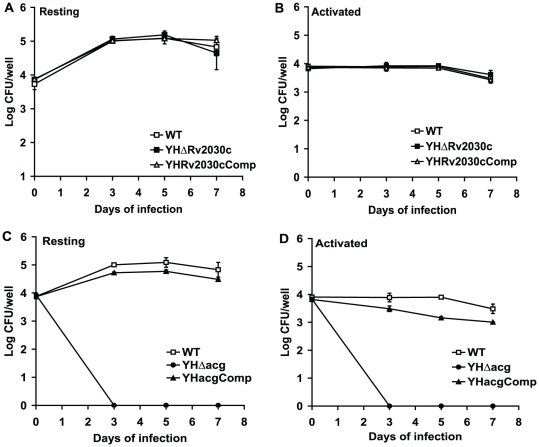
Growth and survival of *M. tuberculosis* YHΔ*acg and* YHΔ*Rv2030c* in macrophages. Infection of YHΔ*Rv2030c* in resting bone marrow derived macrophages (A) and in IFNγ activated bone marrow derived macrophages (B). Infection of YHΔ*acg* in resting bone marrow derived macrophages (C) and in IFNγ activated bone marrow derived macrophages (D). These results are the means and standard deviation derived from one representative of three independent experiments.

We examined the ability of both YHΔ*acg* and YHΔ*Rv2030c* to infect and proliferate within bone marrow derived macrophages from BALB/c mice. As shown in Fig. 2A and 2B, the growth of WT and YHΔ*Rv2030c* in both resting and activated macrophages was similar over the 7 days of infection. These data indicate that the loss of *Rv2030c* has no detectable effect on intracellular survival and proliferation. Although YHΔ*acg* was able to invade macrophages as seen in Fig. 2C and 2D showing similar CFU counts to the WT strain recovered from the macrophages at time 0, the mutant was unable to grow and survive in both resting and activated macrophages. After three days of infection, the entire macrophage lysates (1 ml/well) were plated on 7H11 agar. No CFU counts of YHΔ*acg* were recovered. Similar profiles of YHΔ*acg* were seen in the macrophage-like cell line J774A.1 (data not shown). These results indicated that *acg* is essential for intracellular survival.

As the *acg* gene has been predicted to encode a nitroreductase in *M. tuberculosis* [13], deletion of the gene might render the bacilli sensitive to macrophage nitrogen intermediates. In order to confirm this, we infected macrophages derived from iNOS knock out mice with *M*. *tuberculosis* WT, YHΔ*acg* and the complemented strains. These macrophages produce reduced levels of nitric oxide [26]. If *acg* was a nitroreductase, the growth of the mutant would be restored to its parental level or to a certain extent to the parental level. However, the YHΔ*acg* mutant failed to grow in macrophages derived from the iNOS KO mice (Fig. S1) showing the similar survival patterns in the macrophages derived from WT mice (Fig. 2C and 2D).

### Growth and survival of YHΔ*acg* and YHΔ*Rv2030c* in mice

**Figure 3:**
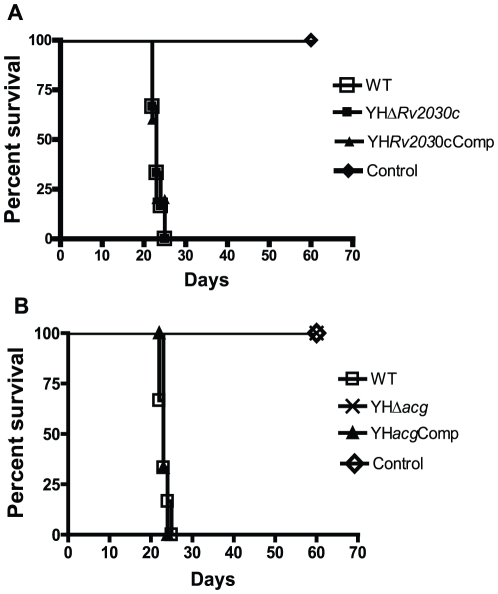
Virulence of *M. tuberculosis* strains in SCID mice. A. WT, YHΔ*Rv2030c* and complemented strain. B. WT, YHΔ*acg,* and complemented strain. Mice (n = 6) were infected intravenously with 10^6^ CFU counts of *M. tuberculosis* H37Rv strains. Survival of SCID mice was observed for 60 days. The results were repeated once with reproducible results. There were significant differences between WT and YHΔ*acg* infected mice (P<0.0001).

In order to verify if the mutants changed their virulence in acute infection in the absence of host acquired immunity, virulence assays of the *M. tuberculosis* strains in immunocompromised SCID mice were carried out as described previously [27]. The mice were infected intravenously with 10^6^ CFU counts of each bacterial strain, and the survival of the SCID mice was observed over 60 days. As shown in Fig. 3A, the mice infected with WT and YHΔ*Rv2030c* survived for 23 and 22.5 days, respectively. In contrast, all mice infected with YHΔ*acg,* like the uninfected control mice, survived for 60 days at which point the experiments were terminated (Fig. 3B). The attenuation of YHΔ*acg* strain was reversed in the *acg* complemented strain (Fig. 3B).

**Figure 4:**
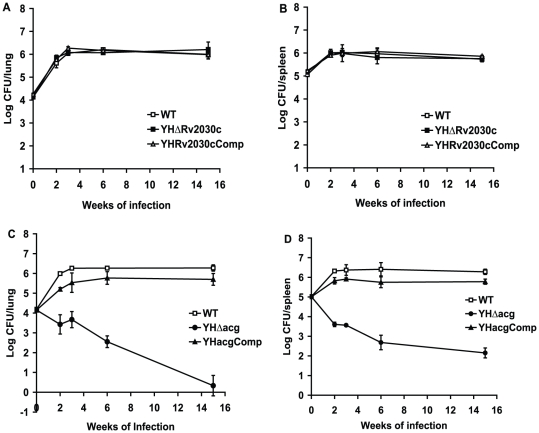
Growth and survival of YHΔ*acg* and YHΔ*Rv2030c* in the lungs and spleen of BALBc mice. Mice were infected with 10^4^ CFU counts of each strain. At different time points the infected mice were sacrificed and the numbers of bacteria in the lungs and spleens were measured. CFU counts in lungs (A) and spleens (B) in mice infected with WT, YHΔ*Rv2030c* and complemented strain. CFU counts in lungs (C) and spleens (D) in mice infected with WT, YHΔ*acg* and complemented strain. The reported values represent the average and the standard deviation obtained for each point from three mice. The experiments have been repeated twice with similar CFU counts in lungs and spleens.

In order to examine the growth and survival of the mutants in the face of host acquired immunity, BALB/c mice were intravenously infected with 1.2×10^4^ CFU counts of the WT and the mutant strains. As shown in Figure 4, typical growth of the WT *M. tuberculosis* H37Rv in the immunocompetent mice was seen. There was exponential growth for approximately three weeks, the CFU counts of the WT reached 10^6^ CFU/lung, followed by a plateau as the acquired immune response inhibited the bacterial growth. The growth of YHΔ*Rv2030c* was similar to that of WT strain (Fig. 4A and 4B). However, after infection with YHΔ*acg* mutant, no growth of the mutant was seen, the CFU counts of the mutant gradually decreased in both lungs and spleens (Fig. 4C and 4D) throughout the course of infection. Almost no *acg* mutant cells were recovered from the infected lungs at 15 weeks of infection which was confirmed by plating the entire lung homogenate on 7H11 agar. Growth characteristics which were similar to WT were seen in the *acg* complemented strain. These data indicate that YHΔ*acg* is attenuated.

### The *acg* mutant stimulated less inflammatory response

**Figure 5:**
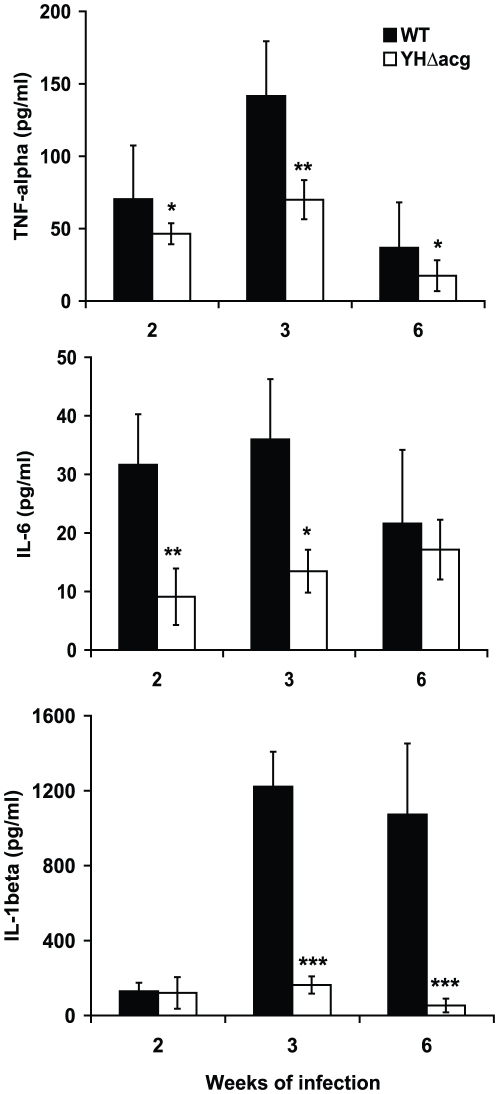
TNF-α, IL-6 and IL-1β levels in BALB/c mice infected with WT and YHΔ*acg* measured by ELISA. The data shown are the averages for the lungs from three mice ± standard errors. Statistical significance was determined by Student's *t* test (*n* = 3) (***, *P*<0.001).

To examine if the mice infected with YHΔ*acg* mutant produced stronger inflammatory response than those infected with the WT strain, which might inhibit the growth of the mutant in the mouse organs, we examined the production of the proinflammatory cytokines TNF-α, IL-6, and IL-1β in the lungs of mice infected with the WT and YHΔ*acg* strains for 2, 3 and 6 weeks by ELISA. As shown in Fig. 5, the mutant stimulated less cytokine production in the host lungs, especially at 2 and 3 weeks of infection for both TNF-α and IL6 and at 3 and 6 weeks for IL-1β.

**Figure 6:**
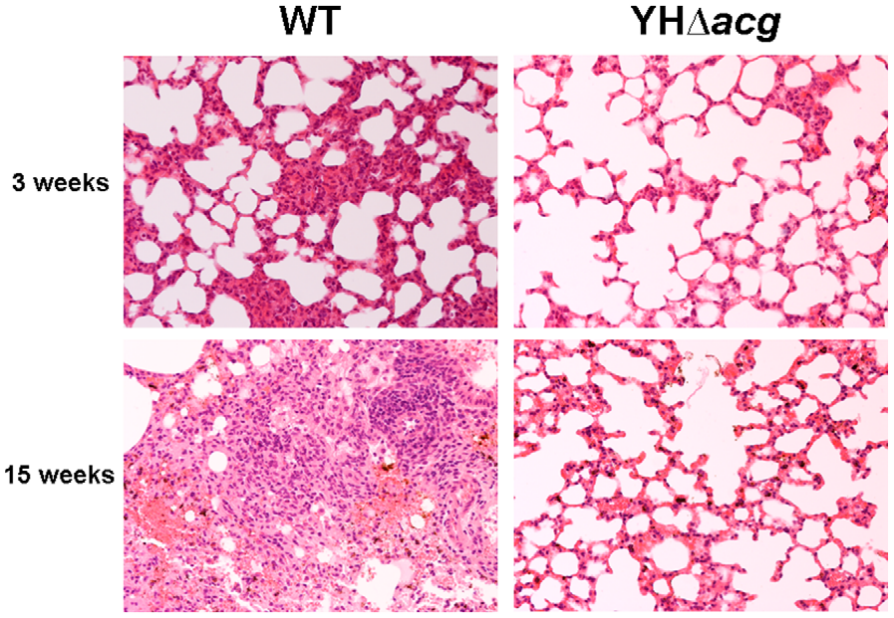
Hematoxylin- and eosin-stained sections of lungs in mice infected with WT and YHΔ*acg* for 3 and 15 weeks. Histopathological examination was carried out using three mice in each group. Two sections from each mouse were examined. The data shown are representative of lung sections from three animals in each experimental group.

The histopathological changes of mice infected with WT and mutant strains were also examined. As shown in Fig. 6, the WT infected mice developed alveolar consolidation at 3 weeks and this was accompanied by the presence of granulomatous inflammation containing lymphoplasmacytic cells, alveolar macrophages, neutrophils, and multinucleated cells at 15 weeks. In contrast, the *acg* mutant infected lungs showed normal lung structure with the alveolar space preserved throughout the lung, indicating that the reduced host immune response and the lack of histopathological changes were due to insufficient bacilli present in the host organs.

### Response to in vitro stress

The WT, YHΔ*acg* and YHΔ*Rv2030c* were grown in 7H9 broth without disturbance for 60 days as described previously [28]. Under these conditions, the bacilli grow in the top layer of the unagitated culture, where oxygen is available. Then they settle to the bottom of the container, where there is a low concentration of oxygen, and slowly adapt to microaerophilic and eventually to anaerobic conditions. Growth characteristics of the mutants were similar to those of the wild-type strain (data not shown), indicating that *acg* and *Rv2030c* genes are not required for the bacterium to grow under *in vitro* hypoxic conditions.

In order to investigate if stress conditions, especially those which *M. tuberculosis* encounters in macrophages, affect the mutants, WT, YHΔ*acg*, YHΔ*Rv2030c* and complemented strains were exposed to hydrogen peroxide (H_2_O_2_), nitric oxide (NO) and acidic conditions. Survival of the strains was examined by CFU counting. No increased susceptibility to NO, H_2_O_2_ and acidic conditions was observed in these mutants in comparison to the WT strain (Fig. S2).

**Figure 7:**
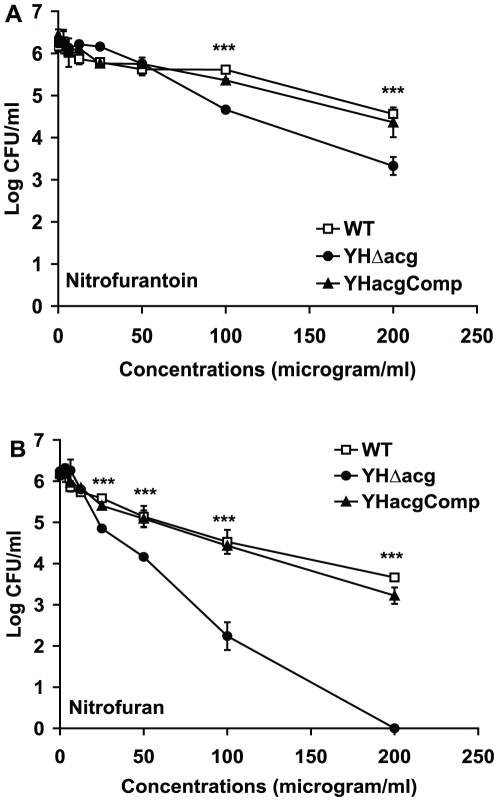
Survival of YHΔ*acg* in response to nitrofuran drugs. The WT, YHΔ*acg* and the complemented strains were treated with nitrofurantoin (A) and nitrofuran (B) at different concentrations for 48 hours. These results are the means and standard deviation derived from one representative of three independent experiments. Statistical significance was determined by Student's *t* test (*n* = 3) (***, *P*<0.001).

In order to investigate if the *acg* gene encoded a nitroreductase, the WT, YHΔ*acg* and the complemented strains were incubated with the antibiotics such as prodrugs nitrofurantoin and nitrofuran, each containing a nitro group in its furan ring. The bactericidal activation of these drugs needs the NAD(P)H dependent nitroreductase. The bactericidal activities of these drugs were examined by CFU counting after two days of incubation with 7 days old cultures. As shown in Fig. 7A, the WT strain was relatively tolerant to nitrofurantoin. At 200 µg/ml, about 1.6 log kill was seen. However, YHΔ*acg* became more sensitive to it than the WT strain; there was about 3 log kill at 200 µg/ml. The mutant was exceptionally sensitive to nitrofuran causing a 6 log kill at 200 µg/ml and a 4 log kill at 100 µg/m (Fig. 7B). The CFU counts of the WT strain were reduced to 2.5 logs and 1.6 logs at 200 and 100 µg/ml, respectively (Fig. 7B). This suggested that Acg protein might not have nitroreductase activity to reduce the nitro group of the drugs; otherwise deletion of the gene should render the bacteria more resistant to the drugs.

### Nitroreductase activity of YHΔ*acg*

**Figure 8:**
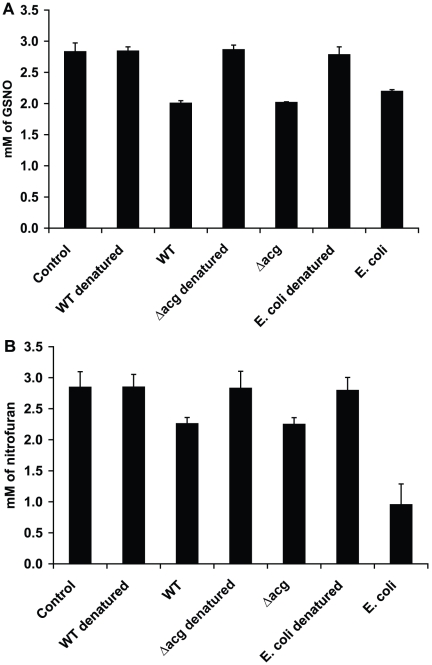
Measurements of nitroreductase activities in YHΔ*acg* using GSNO (A) or nitrofuran (B) as a substrate. Presence of nitroreductase activities was measured by the reduction of the substrate using the bacterial cell lysates at 24 hours for GSNO and 48 hours for nitrofuran. The cell lysate extracted from *E. coli* was added as a positive control. The denatured cell lystaes which were produced by boiling the cell lysates at 100°C for 10 minutes were included as the negative control. The experiments were repeated twice with the similar results.

In order to investigate if the *acg* mutant contained a nitroreductase activity, the nitroreductase activities of the WT and YHΔ*acg* lysates were measured using GSNO or nitrofuranoin or nitrofuran as a substrate. The Acg protein might be contained in the cell lysate as expression of recombinant *M. tuberculosis* Acg in *E. coli* produced a soluble protein (communication with Nicolas Keep). As seen in Fig. 8A, addition of WT and YHΔ*acg* lysates significantly reduced the level of GSNO. However, there is no significant difference between WT and YHΔ*acg* in term of nitroreductase activity (p>0.5). The reduction of GSNO was confirmed by using *E. coli* cell lysate. No nitroreductase activities were observed using denatured cell lysates from the WT, YHΔ*acg* and *E. coli*. As seen in Fig. 8B, there was a reduction of the drug followed by addition of WT, YHΔ*acg* and *E. coli* proteins. Again, there was no difference between the WT strain and the mutant in reduction of nitrofuran. The same results were observed using nitrofurantoin as a substrate (data not shown).

## Discussion

*M. tuberculosis* virulence is measured by the ability of the bacterium to invade, grow and persist in macrophages. This infection causes host tissue damage which is characterised by the formation of a compact cluster of cells, called a granuloma, around the foci of infection. Here we demonstrate that the deletion of *acg* yields an attenuated mutant which fails to grow and persist in macrophages and in immune deficient and immune competent mice. It also showed a deficit in causing tuberculosis granulomatous disease. These data demonstrate that *acg* is an essential factor for *M. tuberculosis* to establish growth in the host cells. Deletion of *Rv2030c* was dispensable to *M. tuberculosis* virulence and persistence in macrophages and in mice.

The *hspX*, *acg* and *Rv2030c* belong to the DosR regulated gene family. Although it has been suggested that DosR is a dormancy survival regulator [6], [23], [29], this supposition is only based upon gene expression under a variety of different stress conditions, in macrophages and in mice. Only a few reports [19] have demonstrated discrete gene functions, for example *hspX*, within the *dosR* regulon using allelic exchange mutants in animal infection models. The differential expression patterns of *hspX* under stress conditions and in vivo may be required to slow the growth of *M. tuberculosis* in order to retain a non-replicating state in latency. This is consistent with the putative function of the *dosRS* regulon. However, the phenotype of the *acg* mutant found in this study suggests that *acg* does not seem to fit into the dormancy associated role of the *dosR* regulon. It is associated with full virulence of *M. tuberculosis* and is required by the bacilli to sustain infection, but not to initiate the infection process because it was able to invade macrophages but unable to grow. *Rv2030c* is directly regulated by *dosR* and is co-transcribed with *hspX*. Surprisingly, deletion of *Rv2030c* has no effect on *M. tuberculosis* growth and persistence in macrophages and in acute and persistent animal models. Recent work suggested that *M. tuberculosis* persistence modulated by *dosR* was regulated by a complex multilayer regulatory network [29]. This was strongly evidenced by the fact that deletion of *hspX* and *acg* resulted in contradicting effects. There is significant discrepancy between *dosR* regulated persistence and the bacterial virulence in mice [22]. This was shown by the generation of a *dosR* mutant which survived normally in mice although the *dosR* regulon was highly upregulated in vivo [22]. Additional evidence which supports this hypothesis comes from the data that suggests *dosR* only controls the initial hypoxic response, whilst enduring hypoxic responses are independent of *dosR* regulation [22]. These results, together with our finding suggest that *dosR* may regulate functionally diverse genes; some are involved in *M. tuberculosis* persistence and others in *M. tuberculosis* virulence or in unknown physiological and pathological roles. In addition, these findings illustrate that information obtained by genome-wide analysis only gives clues as to the function of genes. Discrepancies between gene function and gene expression patterns can only be resolved by other techniques such as gene inactivation.

How does *acg* contribute in *M. tuberculosis* growth and survival in vivo? As described previously [13], [24], sequence homology analysis suggests that *acg* belongs to a nitroreductase family which consists of oxygen-insensitive flavin-dependent enzymes that reduce nitrosubstituted compounds such as nitroaromatics quinones, and riboflavin derivatives to either a hydroxylamino- or aminoaromatic endproduct in a FMN-, FAD-dependent or NAD(P)H dependent manner [30]. As *acg* is upregulated in response to NO, in macrophages [13] and in mice [11], it has been predicted that the product of *acg* may be responsible for *M. tuberculosis* detoxification of nitroaromatic compounds which may be present within macrophages or granulomas [13]. Our results indicated that the *acg* mutant was not able to grow and survive in macrophages which produce reactive oxygen and nitrogen intermediates, acidic conditions and lytic enzymes. However, unlike other *M. tuberculosis* mutants which lack the oxidative and nitrosative detoxification genes such as *ahpC* [31] or *tpX* [26], deletion of *acg* did not compromise the bacterium to grow and survive in acidic, nitric oxide and hydrogen peroxide environments in vitro. Furthermore, the growth and survival of the mutant was not restored in macrophages isolated from iNOS knockout mice. This was confirmed by the fact that *acg* mutant contained the same level of nitroreductase activity to reduce GSNO as the wild type (Fig. 8). It is not known if the *acg* gene product possesses a nitroreductase activity as we are not able to detect it using in vitro methods or using iNOS deficient mice. Further work is underway in our laboratory to study the enzymatic activity of the recombinant Acg protein to metabolise nitroaromatic compounds. Surprisingly, YHΔ*acg* was more sensitive than the WT strains to nitrofurantoin and especially nitrofuran. Nitrofurans are prodrugs which exhibit antimicrobial activity against *Escherichia coli* and *Staphylococcus saprophyticus* as well as other facultative anaerobes, anaerobic bacterial and protozoan species [32], [33]. The antimicrobial activities of these prodrugs are largely dependent on the rapid reduction of the 5-nitro groups by a wide range of enzymes including the NAD(P)H nitroreductases produced by these bacteria, which leads to accumulation of redox active intermediates, including hydroxylamine adducts which cause lethality to the bacterial cells. Nitrofurantoin has a relatively high MIC against *M. bovis* BCG (12 µg/ml) [34] and *M. tuberculosis* H37Rv (49.5 µg/ml) [35]. *M. tuberculosis* is relatively tolerant to nitrofurantoin and nitrofuran showing 1.6 and 2.5 log killing at 200 µg/ml, respectively (Fig. 7). We showed that both the WT and YHΔ*acg* exhibited low activities to reduce nitrofuran drugs (Fig. 8). However, YHΔ*acg* became more sensitive to the drugs. This indicated that the nitrofuran drugs might function in unknown mechanisms in *M. tuberculosis* which differed from the activation of the drugs by reduction of the nitro-group. Further investigation is underway in our laboratory.

In summary, this work demonstrates that one of the *M. tuberculosis dosR* regulated genes, *acg,* is associated with *M. tuberculosis* virulence. The *acg* mutant is unable to grow and survive in macrophages and mice. This attenuation is not associated with a deficiency in response to oxidative and nitrosative stresses. Our data suggest that DosR may control an array of genes which shows functional diversity in vitro, in macrophages and in mouse infections. We have identified a key target against which new anti-TB drugs could be designed, or with which a live vaccine could be developed to prevent *M. tuberculosis* infection.

## Materials and Methods

### Ethics Statement

All animal experiments were conducted according to the Animals Scientific Procedures Act, 1986 (an Act of the Parliament of the United Kingdom 1986 c. 14) (Home Office Project licence Number 70/5933) with approval from St George's, University of London ethics committee. The animal husbandry guidelines and animal procedure for this study were followed according to the Animals Scientific Procedures Act, 1986. The sacrifice of the mice was performed in accordance with humane end point protocols under the Animals Scientific Procedures Act, 1986.

### Bacterial strains and growth conditions

*M. tuberculosis* strain H37Rv was used as a parental strain to construct the mutants. *M. tuberculosis* strains were grown in 7H9 medium supplemented with 10% albumin dextrose complex (ADC, Becton Dickinson) and 0.05% Tween 80, or on 7H11 agar medium supplemented with oleic albumin dextrose complex (OADC, Becton Dickinson). *E. coli* XL2 was used as a host strain for cloning and plasmid propagation in or on liquid and solid Luria-Bertani medium. Antibiotics were used as follows: ampicillin (Sigma) 100 µg/ml, kanamycin (Sigma) 20 µg/ml, gentamicin (Sigma) 20 µg/ml and hygromycin (Invitrgen) 100 µg/ml.

### Mutant construction

The mutant construction was carried out in a 2-step strategy using the pNIL/pGOAL plasmids as described previously [25]. DNA manipulations including DNA isolation, ethanol precipitation of DNA, electrophoresis of DNA in agarose and transformation were performed by standard procedures [36]. Enzyme reactions were performed according to the manufacturer's instructions (Invitrogene and New England Biolabs). To construct *M. tuberculosis acg* deletion, a 2949 bp PCR product containing the *acg* gene (996 bp) and about 1 kb flanking sequences adjacent to each end of the gene was amplified using *M. tuberculosis* H37Rv genomic DNA as template and primers *acg*1 (5′- AATAAGCTTAGCAGTTCCCGACCCTCAC-3′) and *acg*2 (5′- AATAAGCTTCGCTATGGAACCGACGTGC -3′). The PCR product was purified from agarose gel using a QIAquick Gel Extraction Kit (Qiagen) and was cloned into the *Hin*d III site of pGEM3Z (Promega) to form pGEM*acg*. A *Nco* I fragment of 777 bp in the *acg* coding region was deleted. The disrupted *acg* gene with the flanking sequences was cloned into the *Hind* III site of p2NIL [25] to make p2NIL*acg*1. A *hyg*-*sacB* marker cassette from the pGOAL 19 [25] was cloned into the *Pac* I site of p2NIL*acg*1 to form the final mutant construct p2NIL*acg*2. To construct *M. tuberculosis Rv2030c* deletion, a 4129 bp PCR product containing the *Rv2030c* gene and 1 kb flanking sequences adjacent to each end of the gene was amplified using *M. tuberculosis* genomic DNA as template and primers *Rv2030c*1 (5′- AATAAGCTTCGACTCCACCAAGTCCCAA -3′) and *Rv2030c*2 (5′- AATAAGCTTGGCTTCCGGGTTAACGATC -3′). The PCR product was cloned into the *Hind* III site of pGEM3Z (Promega) to form pGEM*Rv2030c*. The *Rv2030c* gene was deleted by PCR with primers *Rv2030c*3 5′- ATTACGCGTAAACCTACCCGACCGGTCT-3′and *Rv2030c*4 (5′- ATTACGCGTACGGACCCAGTGGTCAG>TT-3′) which were designed outwardly starting from the start and stop codons of the *Rv2030c* gene using pGEM*Rv2030c* as a template. The PCR product which contains only the flanking sequences of *Rv2030c* gene was cut with *Mlu* I and ligated to form pGEM*Rv2030c*Δ. The *Rv2030c* flanking sequences containing no *Rv2030c* gene was cloned into the *Hind* III site of p2NIL [25] to form p2NIL*Rv2030c*Δ1. Finally, a *hyg*-*sacB* marker cassette from the pGOAL 19 [25] was cloned into the *Pac* I site of p2NIL*Rv2030c*Δ to form the final mutant construct p2NIL*Rv2030c*Δ2. Plasmid DNA was isolated with a QIAprep Spin Miniprep Kit (Qiagen). The sequences of purified PCR products were determined commercially (QIAGEN). Both DNA strands were sequenced by using the two primers that were used to generate each PCR product. The plasmids p2NIL*acg*2 and p2NIL*Rv2030c*Δ2 were electroporated into *M. tuberculosis* H37Rv cells, respectively, and the selection of the mutants was performed as described previously [25], [37].

To complement the *acg* deletion, a 1395 bp DNA fragment containing the *acg* gene and 324 bp of upstream sequence was amplified by PCR using primers *acg*C1 5′- AATAAGCTTTCCAGCCGCATCAACCGGGT-3′) and *acg*C2 (5′- AATAAGCTTTCGCCGGATCCGCTCATCGA -3′). The PCR product was cloned into the *Hin*d III sites of the integrating plasmid pUC-Gm-Int [38]. For complementation of *Rv2030c* mutant, a 2796 bp PCR product amplified using primer *Rv2030c*5 (5′- ATTTCTAGAGTCACCATGGTGTCCGGCAT-3′) and *Rv2030c*6 (5′-ATTTCTAGATCCAAGGCGGGGTTCATGGT-3′) containing 216 bp of the upstream sequence of *hspX* gene, *hspX* gene and *Rv2030c* was cloned into the *Hind* III sites of pUC-GM-Int. The constructs of complementation were electroporated into YHΔ*acg* and YHΔ*Rv2030c* mutants, respectively, and Gm^R^ transformants were selected.

### Estimation of viability under in vitro stress conditions

*M. tuberculosis* strains were grown in 7H9 medium containing 0.05% Tween 80 supplemented with 10% ADC for 10–15 days. A series of 10 ml standing cultures were used for the determination of CFU counts after exposure to stress. For oxidative stress, hydrogen peroxide (H_2_O_2_) (5 mM and 10 mM) was added to the cultures. For nitric oxide (NO) stress, diethylenetriamine/nitric oxide adduct (DETA/NO) and S-Nitrosoglutathione (GSNO) were added to the cultures at the final concentrations of 5 and 10 mM, and the cultures were incubated at 37°C. For treatment with drugs containing a nitro group, nitrofurantoin and 2-nitrofuran were incubated with cultures at the final concentrations of 200, 100, 50, 25, 12.5, 6.25, 3.125 and 0 µg/ml. For acidic stress, the culture medium was replaced with acidic 7H9 medium (pH 4). CFU counts of the treated cultures and the non-treated cultures were determined at 0 and 7 days (acidic conditions), at 0 and 24 hours (H_2_O_2_, DETA/NO and GSNO) or 0 and 48 hours (nitrofurantoin and nitrofuran). Each stress treatment was carried out in triplicate.

### Nitroreductase assay

Nitroreductase activity was measured using cell lysates of WT, YHΔ*acg* and *E. coli* K12 with GSNO, 2-nitrofuran or nitrofurantoin as a substrate. *M. tuberculosis* strains were grown in 7H9 medium containing 0.05% Tween 80 supplemented with 10% ADC without disturbance for 7 days. *E. coli* was grown in nutrient broth overnight at 37°C with constant shaking at 120 rpm. Bacterial numbers of the cultures were determined by optical density reading at 600 nm and CFU counts. The cultures were washed three times in chilled 50 mM Tris-HCI (pH 7.5) and were then resuspended in the same buffer. The cell suspension was transferred into 2 ml tubes each containing 75 to 150 µm glass beads and lysed by homogenisation using a FastPrep Instrument (Fisher Scientific) for 40 seconds at 6.5 speed. The cell debris was removed by centrifugation at 13,000 rpm for 20 minutes at 4°C followed by filtration. Total protein concentrations of the cell lysates were determined with the Bio-Rad protein assay using bovine serum albumin as a standard. The reduction of GSNO or nitrofuran drugs was determined by a decrease in the absorbance at 335 nm for GSNO (molar extinction coefficient 586 M^−1^cm^−1^) [39], at 373 nm for nitrofurantoin (molar extinction coefficient of 2.63×10^4^ M^−1^cm^−1^) [40] and at 340 nm for 2-nitrofuran. The reaction mixture (1 ml) consisted of enzyme preparation, 50 mM Tris-HCl (pH 7.0), 5 mM GSNO or 0.5 mM nitrofurantoin and 2-nitrofuran, and 0.1 mM NADPH. Equal amounts of the WT and the mutant proteins were used in each preparation.

### Mouse infection models

BALB/c mice (6 to 8 weeks old, female, body weight 18–20 g) were used (Harlan UK Ltd). *M. tuberculosis* cells were resusupended in phosphate-buffered saline (PBS) and were intravenously injected into the mouse with 1.2×10^4^ CFU of the bacterial cells. At different time points, spleens and lungs from 4 mice were removed rapidly after sacrifice and a sterile autopsy was performed. The organs were transferred into 2 ml tubes each containing 1 ml sterile distilled water and 2 mm diameter glass beads. Lungs and spleens of the mice were homogenised using a reciprocal shaker (Thermo Hybaid Ltd) for 40 seconds at 6.5 speed. CFU counts from each lung and spleen were performed using serial dilutions of the homogenates.

Virulence assays of the *M. tuberculosis* strains with SCID mice (6 to 8 weeks old, female, Harlan UK Ltd) were carried out as described previously [27]. 9 mice in each group were intravenously infected with 10^6^ CFU of the WT, mutant and the complemented strains. After 4 hours of infection, three mice in each group were sacrificed and CFU counts in lungs and spleens were estimated. The remaining 6 infected mice were observed for 60 days and the death time for each group was recorded. Median survival times were calculated for each group, and statistical analysis was performed using the log rank tests of survival.

### Macrophage infection

Bone marrow-derived macrophages were isolated from BALB/c, C57BL/6 and the iNOS KO (Inducible Nitric Oxide Synthase Knockout) mice and cultured for 7 days as described previously [27]. Also macrophage J774A.1 cell line was used. Adherent macrophages were harvested and seeded at 2×10^5^ cells per well of 24 well plates in Dulbecco's Modified Eagle Medium (Invitrogen) without L-cell conditioned medium, penicillin and streptomycin. Activation of macrophage cells was carried out by the addition of interferon-γ (IFN-γ, 100 U/ml. R&D Systems) for 24 h, followed by the addition of lipopolysaccharides (LPS, 200 ng/ml, Sigma) for 3 h. The cells were infected with the bacterial strains at a multiplicity of infection of 1∶1 for 4 hours, then washed 6 times with warm Hank's Buffered Salt Solution (HBSS). At 0, 3, 5 and 7 days after infection, the cells were washed and lysed with 0.1% Triton-X 100, and CFU counts were performed. At each time point, the infected macrophages in two wells were individually harvested using Trypsin-EDTA and stained for acid-fast bacilli (AFB) in order to check macrophage viability. The experiments were carried out three times in triplicate.

## Supporting Information

Figure S1 **Growth and survival of YHΔ** ***acg*** **in resting and IFN-γ-activated macrophages derived from iNOS knockout mice.** These results are the means and standard deviation derived from one representative of three independent experiments.
(EPS)

Figure S2 **Survival of YHΔ** ***acg*** **and YHΔ** ***Rv2030c*** **in response to DETA/NO at 5 and 10 mM (A), H_2_O_2_ at 5 and 10 mM (B), and under acidic condition (C).** The data shown is a representative of three independent experiments. Data are represented as mean ± SD of triplicate tests.
(EPS)
